# Understanding the promoting effect of non-catalytic protein on enzymatic hydrolysis efficiency of lignocelluloses

**DOI:** 10.1186/s40643-021-00363-9

**Published:** 2021-01-29

**Authors:** Zhenggang Gong, Guangxu Yang, Junlong Song, Peitao Zheng, Jing Liu, Wenyuan Zhu, Liulian Huang, Lihui Chen, Xiaolin Luo, Li Shuai

**Affiliations:** 1 College of Materials Engineering, Fujian Agriculture and Forestry University, Fuzhou, 350002 China; 2 Jiangsu Provincial Key Laboratory of Pulp and Paper Science and Technology, Nanjing Forestry University, Nanjing, 210037 China; 3 Department of Materials Science & Engineering, Southern University of Science and Technology, Shenzhen, 518055 China

**Keywords:** Enzymatic hydrolysis, Peanut protein, Lignin deposits, Adsorption, Blocking, Pretreatment

## Abstract

**Abstract:**

Lignin deposits formed on the surface of pretreated lignocellulosic substrates during acidic pretreatments can non-productively adsorb costly enzymes and thereby influence the enzymatic hydrolysis efficiency of cellulose. In this article, peanut protein (PP), a biocompatible non-catalytic protein, was separated from defatted peanut flour (DPF) as a lignin blocking additive to overcome this adverse effect. With the addition of 2.5 g/L PP in enzymatic hydrolysis medium, the glucose yield of the bamboo substrate pretreated by phenylsulfonic acid (PSA) significantly increased from 38 to 94% at a low cellulase loading of 5 FPU/g glucan while achieving a similar glucose yield required a cellulase loading of 17.5 FPU/g glucan without PP addition. Similar promotion effects were also observed on the n-pentanol-pretreated bamboo and PSA-pretreated eucalyptus substrates. The promoting effect of PP on enzymatic hydrolysis was ascribed to blocking lignin deposits via hydrophobic and/or hydrogen-bonding interactions, which significantly reduced the non-productive adsorption of cellulase onto PSA lignin. Meanwhile, PP extraction also facilitated the utilization of residual DPF as the adhesive for producing plywood as compared to that without protein pre-extraction. This scheme provides a sustainable and viable way to improve the value of woody and agriculture biomass.

Peanut protein, a biocompatible non-catalytic protein, can block lignin, improve enzymatic hydrolysis efficiency and thereby facilitate the economics of biorefinery.

**Graphical abstract:**

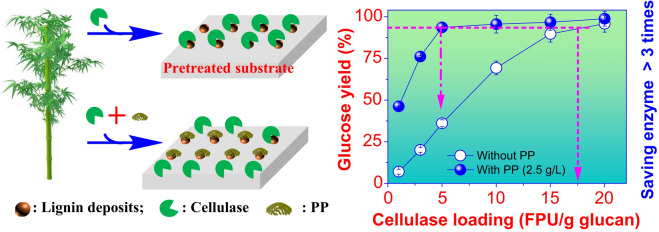

## Introduction

Lignocelluloses-derived glucose is an important platform molecule for producing biofuels and chemicals (Ragauskas et al. 2006). Hydrolysis of cellulose with cellulolytic enzymes (or cellulases) as biological catalysts is a promising method for deconstructing cellulose to fermentable glucose (Shuai et al. 2016b). However, the complex and compact structure of lignocelluloses formed from cellulose, hemicellulose, and lignin makes cellulose inaccessible to cellulolytic enzymes (Himmel et al. 2007). To increase cellulose accessibility, a pretreatment process prior to enzymatic hydrolysis is necessary to partially or fully remove the recalcitrance of woody biomass such as lignin and hemicelluloses to expose cellulose (Öhgren et al. 2007; Yang and Wyman 2004). Many pretreatment methods such as traditional dilute acid (DA) (Sun and Cheng 2005) as well as acidic organosolv [such as ethanol (ET)] (Pan et al. 2005) and hydrotrope [such as phenylsulfonic acid (PSA)] (Chen et al. 2017; Luo et al. 2020) solvent pretreatments have been widely studied. DA pretreatment removes a limited amount of lignin from lignocellulosic biomass and the residual lignin within cell wall undergoes the processes of melting, aggregating, and/or redistributing to expose cellulose for enzymatic hydrolysis (Donohoe et al. 2008; Selig et al. 2007). Hydrotrope and organic solvent-induced pretreatments (such as PSA and alcohol-based pretreatments) can remove most of lignin from woody biomass (Bian et al. 2017; Chen et al. 2017; Shuai et al. 2016a), but the dissolved lignin could be re-deposited on the surface of the pretreated solid substrates during cooling and/or washing processes (Liu et al. 2019; Luo et al. 2020; Shi et al. 2019). These lignin deposits could adsorb cellulase non-productively, leading to the reduction of enzymes involved in enzymatic hydrolysis and thereby reduced hydrolysis rates of cellulose in pretreated substrates. Due to the non-productive adsorption of enzymes to lignin deposits, high enzyme loadings are required to achieve decent glucose yields for these pretreated substrates. For example, even though 92% of lignin in poplar wood was removed by *p*-toluenesulfonic acid pretreatment, a cellulase loading as high as 15 FPU/g glucan was essential for achieving a 90% glucose yield of the pretreated substrate (Chen et al. 2017). High dependence on costly enzymes during enzymatic hydrolysis would certainly raise the cost of a biorefining process (Klein-Marcuschamer et al. 2012).

Currently, developing lignin-blocking additives has been regarded as a promising method for overcoming the adverse effect of lignin on enzyme hydrolysis, in addition to the enzyme engineering and genetic modification of lignin (Li and Zheng 2017; Liu et al. 2016). A lignin-blocking additive can be adsorbed onto lignin and reduce the non-productive adsorption of cellulase onto lignin. Reported lignin-blocking additives mainly include polymeric additives, non-catalytic proteins, and metal ions (Li and Zheng 2017; Liu et al. 2016; Saini et al. 2016). Polymeric additives such as Tween series (Eriksson et al. 2002), polyethylene glycol (PEG) (Börjesson et al. 2007), polyvinylpyrrolidone (PVP) (Cai et al. 2017a) and lignin derivatives (such as lignosulfonates and lignin-based amphiphilic compounds) (Cai et al. 2017b; Wang et al. 2013) could be adsorbed onto lignin or enzymes through hydrophobic and/or electrostatic interactions and the non-productive adsorption of enzymes to lignin would be significantly alleviated due to the electrostatic repulsion and/or steric hindrance of the additives adsorbed onto lignin (Li and Zheng 2017; Liu et al. 2016). Some metal ions (such as Ca^2+^ and Mg^2+^) could form complexes with lignin, which can reduce the active sites (such as phenolic hydroxyl groups) or surface charge of lignin and thereby weaken the hydrogen-bonding and electrostatic interactions between lignin and enzymes (Akimkulova et al. 2016; Liu et al. 2010). Although polymeric additives and metal ions can effectively promote enzymatic hydrolysis, both of them would cause additional issues to downstream fermentation microorganism, product separation, and waste liquor treatment (Li and Zheng 2017; Liu et al. 2016; Saini et al. 2016). Non-catalytic proteins [such as bovine serum albumin (BSA) and casein] are biocompatible (Kristensen et al. 2007), but the preparation and purification processes of BSA and casein are costly. The development of inexpensive non-catalytic proteins as lignin-blocking additives will be a viable solution to reduce the loading of costly enzymes and stimulate the development of glucose-based biorefineries. Meanwhile, understanding the lignin-blocking mechanism will provide guidance for exploring alternative proteins for such an application (Florencio et al. 2016; Luo et al. 2019).

**Fig. 1:**
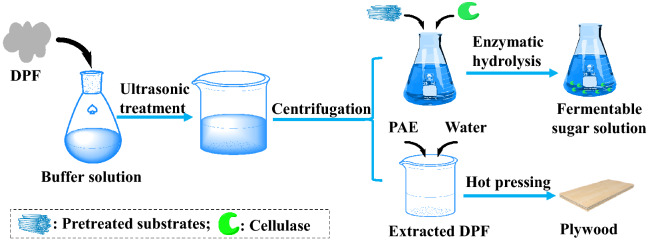
Schematic diagram of using DPF to prepare lignin-blocking additive and bio-adhesive

In this article, a non-catalytic protein, peanut protein (PP), was easily isolated from inexpensive and abundant defatted peanut flour (DPF) and used as a lignin-blocking additive to promote the enzymatic hydrolysis of bamboo and eucalyptus substrates pretreated by various acidic pretreatments while the resultant residue of DPF was further processed as a bio-adhesive (Fig. 1). We systematically evaluated the performance of PP for promoting enzymatic hydrolysis of solid substrates resulting from different pretreatment methods and analyzed the mechanism of PP for blocking lignin. This study can not only serve as a potential process for improving the economics of lignocellulosic biorefinery, but also provide a viable way to improve the overall utilization value of DPF.

## Materials and methods

### Materials

Chemicals including phenylsulfonic acid (PSA), poly(vinyl alcohol) (PVA), and Tween 20 were purchased from Aladdin® Chemicals (Shanghai, China). Cellulase (Celluclast1.5 L®, 51 FPU/mL), β-glucosidase (Novozyme 188, 413 CBU/mL), Avicel (Avicel® PH-101), lignosulfonate (LS), N,O-Bis(trimethylsilyl)trifluoroacetamide and pyridine were purchased from Sigma-Aldrich Company (Shanghai, China). BSA and Pierce™ BCA Protein Assay Kit were ordered from VWR™ (Shanghai, China) and Thermo-Fisher Scientific Life (Rockford, IL, USA). Bamboo and eucalyptus particles (40 − 60 mesh), polyamide-polyamine epichlorohydrin (PAE) resin and DPF (passed 80-mesh sieve) were provided by Qingshan Paper Co., Ltd. (Sanming City, China), Qingzhou Jinhao Industry & Trade Co., Ltd. (Qingzhou, Shandong, China) and Tianshen Bioprotein Co., Ltd. (Linyi city, China), respectively. All chemicals were analytical grade and used as received.

### Acidic pretreatments

PSA pretreatments were conducted in a three-necked round-bottom flask equipped with a condenser and a thermometer. After bamboo or eucalyptus particles and fresh pretreatment liquor were added, the flask was placed in a glycerin bath for heating. The ratio of pretreatment liquor to wood particle (o.d.) was 15:1 (w/w). The fresh pretreatment liquor was prepared by dissolving PSA in deionized (DI) water at 40 − 60 °C to make the concentration of PSA 80 wt%. The solid–liquid mixture in the flask was magnetically stirred and heated at 95 °C for 30 min. At the end of the pretreatment, the flask was quickly taken off the glycerin bath and cooled to room temperature with tap water. The pretreated slurry was then separated by vacuum filtration and the solid residue after filtration was thoroughly washed with DI water until the pH of the filtrate to be 6−7. The pretreatment liquor and solid substrates were saved for various analyses indicated below and enzymatic hydrolysis.

Diluted acid (DA), ethanol (ET), and n-pentanol (PTL) pretreatments were conducted in a microwave reactor (XH-800G, Xiang Hu Technology Co., Ltd., Beijing City, China). DA pretreatment was conducted at 175 °C for 30 min with a sulfuric acid loading of 2 wt% (on o.d. bamboo particles). The ratio of water to bamboo particles (o.d.) for DA, ET and PTL pretreatments were 6:1 (v/w). For ET and PTL pretreatments, corresponding pretreatment conditions are listed in Table 1. After each pretreatment, the pretreated solid substrate and pretreatment liquor were collected with the same procedure as that of PSA pretreatment.

**Table 1:** The contents and removal of main components in solid substrates pretreated by DA and PSA pretreatments

Substrates^a^	Temp. (°C)	Time (min)	Organic solvent charge (%)	Sulfuric acid charge (%)	Solid substrate yield (%)	Components content/removal (%)^b^		
						Glucan	Xylan	Lignin
Un-Ba						40.2	23.7	26.8
DA-Ba	175	30	NA	2	83.5	45.9/4.7	18.7/34.1	28.6/10.9
PSA-Ba	95	30	80	NA	43.6	83.4/9.6	6.9/87.3	5.7/90.7
ET-Ba	140	20	80	4	66.4	58.5/3.4	18.1/49.2	21.5/46.7
PTL-Ba	130	20	80	4	43.3	84.2/9.3	4.7/91.4	6.9/88.9
Un-Eu						45.2	21.5	21.9
PSA-Eu	95	30	80	NA	45.4	85.6/14.0	6.4/93.6	6.6/86.3

^a^Un-Ba and Un-Eu represent untreated bamboo and eucalyptus particles; PSA-Ba, ET-Ba, DA-Ba, PTL-Ba, PSA-Eu are the abbreviations of phenylsulfonic acid (PSA), ethanol (ET), dilute acid (DA) and n-pentanol (PTL) pretreated bamboo (Ba), and phenylsulfonic acid (PSA) pretreated eucalyptus (Eu) substrates, respectively

^b^The data before slashes are the contents of the components in raw materials (o.d.) and pretreated substrates (o.d.), while the data after slashes refer to the removal (Eq. (1)) of specific component

The composition of the raw material and pretreated solid substrates were measured according to a well-established protocol developed by US National Renewable Energy Laboratory (Sluiter et al. 2012). The removal of each component was calculated by the following equation:

1 $$R = \frac{{100\left( {C_{1} - C_{2} \times Y} \right)}}{{C_{1} }},$$

where *R* is the component removal (%) that was calculated based on the content of the specific component (glucan, xylan or Klason lignin) in the raw materials (o.d.); *C*_1_ and *C*_2_ are the contents (%, on the o.d. basis) of specific component in raw material (o.d.) and pretreated substrate (o.d.), respectively; *Y* (%, on the basis of the o.d. raw material) is the solid substrate yield after pretreatment.

### PP extraction

Water-soluble PP was extracted from DPF according to a reported method (Luo et al. 2019). In brief, DPF was mixed with acetate (1:5, w/v) buffer solution (50 mmol/L, pH 5.0) and magnetically stirred at 150 rpm and 25 °C for 2 h. The slurry was then ultrasonically treated at 300 W for 30 min. The supernatant containing PP was obtained by centrifuging the slurry at 5000 rpm for 5 min. A BCA (bicinchoninic acid) Protein Assay Kit was used to measure the protein concentration in the supernatant (Luo et al. 2019; Smith et al. 1985). The extracted solution was directly used for enzymatic hydrolysis without additional concentration or dilution. For comparison, peanut protein isolate (PPI) was also isolated by a traditional alkali dissolution–acid precipitation method (Zheng et al. 2015).

### Enzymatic hydrolysis

The solid substrate containing glucan used for enzymatic hydrolysis was firstly mixed with acetate buffer solution (50 mmol/L) at a 2% (w/v) solid loading in an Erlenmeyer flask. To allow the sufficient interaction of lignin-blocking additive with lignin, additive (PP, LS, PVA, Tween 20 or BSA) was added 2 h earlier than enzymes for all enzymatic hydrolysis experiments. After adding enzymes, the sealed flasks would be kept in a shaker (MaxQ 481 HP, Thermo-Fisher Scientific, MA, USA) with a shaking speed of 150 rpm for 72 h at 50 °C. Samples were collected at 72 h to determine the concentration of released glucose. In addition to the PP supernatant, other lignin-blocking additives (LS, PVA, Tween 20 and BSA) were initially dissolved or diluted by acetate buffer solution (pH 5.0, 50 mmol/L) prior to being added to the flask. The concentration of lignin-blocking additives in enzymatic hydrolysates was 0–3.5 g/L. Cellulase loadings were 1–20 FPU/g glucan; the pH value of the buffer solution for these hydrolysis experiments was 4–6. For each enzymatic hydrolysis experiment, the amount of β-glucosidase (CBU/g glucan) was twice the cellulase loading (FPU/g glucan). To test the performance and adaptability of PP as a lignin-blocking additive, bamboo raw material pretreated with different pretreatments (PSA and DA) were tested. For comparison, pure cellulose (Avicel) was also used as a control substrate, respectively. The concentration of glucose in the enzymatic hydrolysates was measured by a glucose analyzer (2900D, YSI Inc., Yellow Springs, OH, USA).

Because the glucose concentration in the PP supernatant can be ignored (Table S1), the glucose yield (%) of a pretreated solid substrate or Avicel after 72-h enzymatic hydrolysis is therefore calculated as:

2 $${\text{Glucose yield}} = \frac{{100\left( {C_{{{\text{glu}}}} } \right) \times V \times 0.9}}{{1000\left( {W \times \eta \div 100} \right)}},$$

where *C*_glu_ is the concentration (g/L) of glucose in the enzymatic hydrolysate after 72-h hydrolysis; *V* is the total volume (mL) of the enzymatic hydrolysate; 0.9 is the conversion factor for glucose to glucan; *W* is the weight (g, o.d.) of a pretreated solid substrate or Avicel used for enzymatic hydrolysis; *η* is the content (%) of glucan in a substrate or Avicel.

### Separation of PSA bamboo lignin and milled bamboo lignin

The PSA pretreatment liquor obtained under the pretreatment condition of 80 wt% PSA at 95 °C for 30 min was diluted 15 times by DI water to precipitate out PSA-pretreated bamboo lignin (abbreviated as PSA bamboo lignin) which was then separated through centrifugation at 10,000 rpm for 10 min (Avanti J-30I, Beckman Coulter Inc., CA, USA). After removing the supernatant, the PSA bamboo lignin in the centrifuge tube was mixed with fresh DI water and then transferred to a dialysis bag (1000 Da molecular weight cut-off) for purification. The slurry was dialyzed until the formation of stable suspension. The suspension was further diluted with acetate buffer solution (pH 5.0, 50 mmol/L) for dynamic light scattering (DLS) and zeta potential measurements. The concentration of lignin in the diluted suspension was determined gravimetrically. The left suspension was freeze-dried and the resultant solid powder was used for other analyses. The milled bamboo lignin (MBL) was separated from the untreated bamboo raw material by a reported two-step enzymatic and acid hydrolysis process (Argyropoulos et al. 2002).

### DLS and zeta potential analyses

The particle sizes and zeta potentials of PSA bamboo lignin suspension, cellulase and PP supernatants as well as cellulase- and PP–PSA bamboo lignin complexes were analyzed by a DLS-zeta potential analyzer (Zetasizer Nano ZS90, Malvern Instruments, Malvern, UK) at 25 °C. To remove cell debris, commercial cellulase preparation was diluted by acetate buffer solution (pH 5.0, 50 mmol/L) and centrifuged at 10,000 rpm for 10 min (Avanti J-30I, Beckman Coulter Inc., CA, USA). Based on the BCA method, the concentrations of proteins in cellulase and PP supernatants were measured to be 123.0 and 60.4 mg protein/L, respectively. To verify the adsorption of protein to lignin, PSA bamboo lignin suspension (6.7 mg/L) and protein (cellulase or PP) supernatant were mixed at 25 °C to make a mixture containing a lignin and protein mass ratio of 1:1, and the mixture was then subjected to DLS analysis immediately. Prior to the zeta potential measurement, the pH values of cellulase and PP supernatants as well as cellulase- and PP–PSA bamboo lignin mixtures were also adjusted to 3–6 by 0.1 mol/L HCl or NaOH solution, respectively.

### Quartz crystal microbalance (QCM) measurement

Prior to the QCM measurement, PSA bamboo lignin was spin-coated on a QCM gold sensor (Västra Frölunda, Sweden) according to previous reports (Cai et al. 2017a, b). A QCM (E4 model, Biolin Corp., Gothenburg, Sweden) was employed to differentiate the adsorption of cellulase and PP on the PSA bamboo lignin films. First of all, the QCM flow module was cleaned by Milli-Q water at a high flow rate (> 0.15 mL/min) and balanced by acetate buffer solution (pH 5.0, a concentration of 50 mmol/L and a flow rate of 0.15 mL/min) at 23 °C. With a stable baseline, cellulase in the acetate buffer solution (pH 5.0, 50 mmol/L) was injected into the QCM flow module at a flow rate of 0.15 mL/min. To investigate the stability of the cellulase adsorbed on PSA bamboo lignin, the cellulase buffer solution was switched back to the fresh acetate buffer solution (pH 5.0, a concentration of 50 mmol/L and a flow rate of 0.15 mL/min) to desorb cellulase once the adsorption on the lignin film reached equilibrium. The fresh acetate flow was stopped when a new equilibrium was reached. The adsorption of PP solution (0.5 g protein/L) onto the lignin film was studied in the same way. Based on the overtones (*n* = 3, 5, 7, 9, 11, and 13) of frequency changes (Δ*f*) monitored, the third overtone was commonly selected to evaluate the adsorption data due to the consideration towards stability and reproducibility. Based on the Sauerbrey equation (Lai et al. 2019; Sauerbrey 1959), the maximum adsorption capacity (MAC, ng/cm^2^) of PP or cellulase on the lignin film was calculated as follows:

3 $${\text{MAC}} = - C\frac{\Delta f}{n},$$

where *C* is the constant (17.7 ng/cm^2^/Hz); Δ*f* is the frequency change (Hz); n is the third overtone (*n* = 3).

### Contact angle measurement

PSA-pretreated bamboo substrate (Table 1) was fully delignified by sodium chlorite/acetic acid co-solvent to obtain holocellulose (Sannigrahi et al. 2011). Holocellulose was further silanized with N,O-Bis(trimethylsilyl)trifluoroacetamide to dissolve holocellulose derivatives in pyridine (Österberg 2000). PSA bamboo lignin was dissolved in pyridine–acetic acid–water (9:1:4 (volume ratio)), and PP was dissolved into acetate buffer solution. Prior to contact angle tests, the resultant PSA bamboo lignin, holocellulose, and PP solutions were spin-coated on a silicon wafer and dried in nitrogen gas atmosphere based on a previous method (Cai et al. 2017a), respectively. After the solvent evaporated, the trimethylsilyl groups of the holocellulose film coated on the silicon wafer were hydrolyzed by hydrochloric acid vapor, soaked in DI water and re-dried in nitrogen gas atmosphere to release hydroxyl groups of carbohydrates (Österberg 2000). The contact angles for all samples coated on silicon films were measured (DSA30S, Krűss GmbH, Germany) using pure water.

### Other characterizations of pretreated substrates, protein and lignin

The bamboo raw material and PSA-Ba substrate were oven dried and then subjected to the characterization of scanning electron microscopy (SEM, JEOL JSM-7500F, Japan). The X-ray photoelectron spectroscopy (XPS, ESCALAB250, Thermo-Fisher Scientific, US) characterization of the bamboo raw material and PSA-Ba substrate, and ^31^P-NMR (Bruker Avance III 400 MHz spectrometer) analysis of lignin samples (PSA bamboo lignin and milled bamboo lignin) were conducted according to a previous report (Liu et al. 2019). An X-ray diffraction (XRD) instrument (XRD-6000, Shimadzu, Japan) equipped with a Cu Kα radiation (*λ* = 0.154 nm) was used to measure the crystallinity index (CrI) of cellulose in the PSA-Ba substrate.

Sodium dodecyl sulphate-polyacrylamide gel electrophoresis (SDS-PAGE) of PP, PPI and BSA was analyzed according to a reported method (Zheng et al. 2015). In brief, a 12% acrylamide resolving gel and a 5% acrylamide stacking gel were used for electrophoresis (Bio-Rad PowerPac, Hercules, CA, USA). After electrophoresis, separated proteins contained in gels were stained by Coomassie Brilliant Blue solution and identified using M5 Prestained Protein Ladder (10–180 kDa, Mei5 Biotechnology, China) as molecular weight markers.

### Bio-adhesive preparation

The DPF-based adhesives and plywood were fabricated according to a reported method (Zheng et al. 2019b). Specifically, the content of non-extracted or extracted DPF in DI water was 15 wt%. After adjusting the pH value to 11, the slurry was further mixed with PAE (3 wt%) to obtain the adhesive that was used to prepare plywood. Insoluble fraction content and swelling of the prepared adhesives were measured according to a reported method (Zheng et al. 2019a). A three-layer plywood (eucalyptus veneers, 10 × 2.5 cm) was prepared at an adhesive spread rate of 280 g/m^2^ followed by hot press at 140 °C under a pressure of 1.2 MPa for 6.75 min. Ten specimens were used to measure the dry and wet strength for each sample. Dry and wet bonding strength of the prepared specimens were tested according to the Chinese National Standards (GB/T 9846-2015) at an Instron 5967 universal testing machine (Instron Corp., MA). For wet bonding strength, the specimens were pre-soaked in water at 63 °C for 3 h, and then dried in air for 10 min prior to the test.

## Results and discussion

### Comparison of different lignin-blocking additives for promoting the enzymatic hydrolysis

The performances of different lignin-blocking additives were evaluated through monitoring the glucose yield of each substrate upon their additions to enzymatic hydrolysis. To facilitate the discussion, bamboo (Ba) particles pretreated by a certain pretreatment (DA or PSA) would be abbreviated as DA-Ba (DA-pretreated bamboo) and PSA-Ba (PSA-pretreated bamboo), respectively (Table 1). With bamboo as the raw material, robust xylan and lignin removal (> 85%, Table 1) were achieved by PSA pretreatment while only 32.9% of xylan and 9.3% of lignin were removed during DA pretreatment. Although higher lignin removal was achieved by the PSA-Ba substrate, more and smaller lignin deposits on the PSA-Ba substrate than those on the DA-Ba substrate were observed (Additional file 1: Fig. S1). This is because dissolved lignin would be easily precipitated out and deposited on the surface of the pretreated substrates during the processes of cooling and washing the solid substrates (Yu et al. 2020).

In order to characterize the lignin deposits, XPS was further used to analyze the contents of C and O and their linkages on the surface of the bamboo raw material and PSA-Ba substrate. After pretreatment, the O/C ratio decreased from 0.56 for bamboo raw material to 0.46 for PSA-Ba substrate, resulting in higher surface lignin coverage (*S*_lig_) of the latter (73%) than that of the former (54%) (Additional file 1: Table S2). Moreover, the Scan A (C–C and C–H bonding bonds, 284.4 eV) of C1s peaks on the surface of PSA-Ba substrate (Additional file 1: Fig. S2; Table S2) was also higher than bamboo raw material. The ^31^P-NMR characterization results (Additional file 1: Fig. S3) further illustrated that the content of condensed phenolic hydroxyl groups on PSA bamboo lignin was twice higher than that on the milled bamboo lignin (Additional file 1: Table S3), whereas the content of aliphatic hydroxyl groups decreased three times after PSA pretreatment. These results indicate that in addition to removing most of lignin from lignocellulose, PSA pretreatment also caused the condensation and re-deposition of lignin. Previous studies (Ko et al. 2015; Li and Zheng 2017; Liu et al. 2016; Saini et al. 2016) found that the non-productive adsorption capacity of condensed lignin to enzymes was significantly higher than that of the original milled wood lignin. It is reasonable to speculate that PSA pretreatment can lead to increasing accessibility of lignocellulose to enzymes by removing non-cellulosic components but deposited lignin, especially condensed lignin, still has adverse effects on the enzymatic hydrolysis (Luo et al. 2020). Lignin deposits could inhibit enzymatic hydrolysis via non-productive adsorption and/or steric hindrance effects (Lan et al. 2020; Yu et al. 2020). Therefore, the integration of an efficient pretreatment and a biocompatible lignin-blocking additive could be a solution to the above problems.

**Fig. 2:**
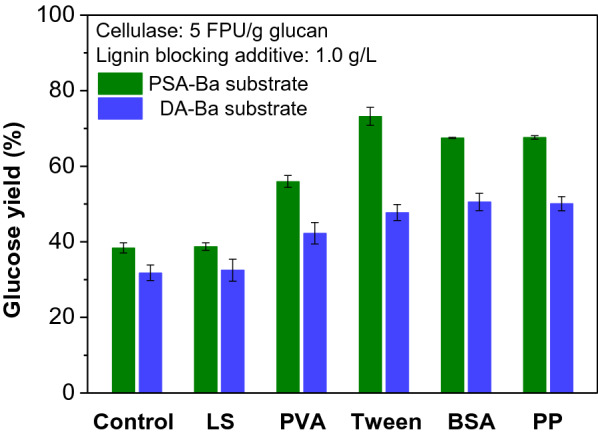
Effects of different lignin-blocking additives on the glucose yields of PSA-Ba and DA-Ba substrates. The addition concentration of lignin-blocking additives, cellulase and β-glucosidase loadings in enzymatic buffer solution were 1.0 g/L, 5 FPU, and 10 CBU/g glucan, respectively. The pH value of enzymatic buffer solution was 5. LS, PVA, Tween, BSA, and PP refer to the lignosulfonate, poly(vinyl alcohol), Tween 20, bovine serum albumin, and peanut protein, respectively

With different contents of residual lignins, the DA-Ba and PSA-Ba substrates were used to evaluate the blocking effects of different additives on lignin deposits during enzymatic hydrolysis. For the same substrate, these lignin-blocking additives showed varied effects on the enzymatic hydrolysis. The glucose yield of PSA-Ba substrate increased from 38.4 to 73.2% with LS, PVA, Tween, BSA and PP as the lignin-blocking additives (Fig. 2). The highest glucose yield (73.2%) was achieved by Tween 20, followed by the non-catalytic proteins (PP: 67.6%; BSA: 67.5%). In contrast, these lignin-blocking additives had limited promoting effects on DA-Ba substrate. The glucose yields of DA-Ba substrate increased by only 59% for BSA (31.8–51.6%), 57% for PP (31.8–50.1%), and 50% for Tween 20 (31.8–47.8%) additions, respectively. These results indicate two key points: (1) Tween 20 and non-catalytic proteins (PP and BSA) are more effective than other lignin-blocking additives used in this study to promote the enzymatic hydrolysis of glucan; (2) the accessibility of glucan in the pretreated substrate to enzyme is also critical to the final glucose yield of each substrate and high residual lignin content would lead to low accessibility of glucan to enzyme.

Non-catalytic proteins are biocompatible with downstream processes such as fermentation as compared to the non-ionic surfactant (e.g., Tween 20) (Li and Zheng 2017; Liu et al. 2016; Saini et al. 2016). In view of the expensive market price of BSA and the impact of residual lignin content on cellulose accessibility, non-catalytic PP (Fig. 1) and PSA-Ba substrate with low residual lignin and high glucan exposure (Table 1) were selected as an efficient lignin-blocking additive and a digestible substrate for further studies.

### Promoting effect of PP on enzymatic hydrolysis

**Fig. 3:**
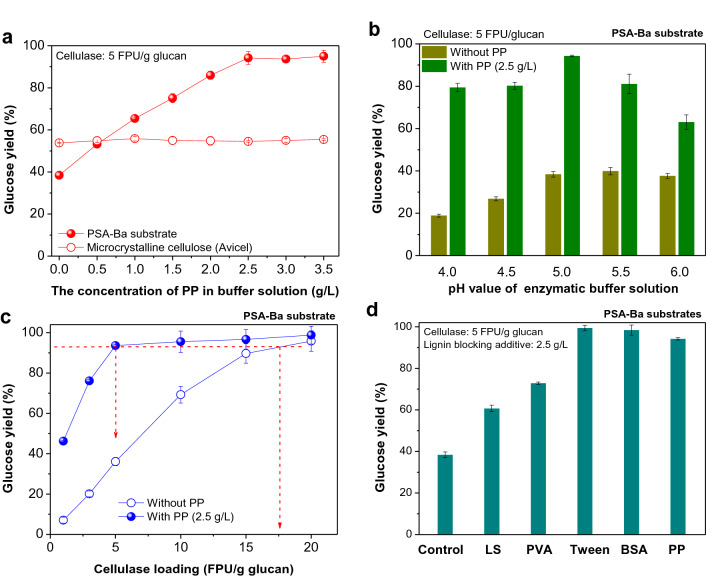
Effects of **a** PP loading, **b** the pH value of buffer solution, **c** cellulase loading and **d** different lignin-blocking additives on the glucose yields of PSA-Ba substrate or microcrystalline cellulose (Avicel). The pH value of the enzymatic buffer solution used in **a**, **c** and **d **was 5. The β-glucosidase loadings used in **a**, **b** and **d** were 10 CBU/g glucan. For each enzymatic hydrolysis experiment conducted in **c**, the amount of β-glucosidase (CBU/g glucan) is twice the cellulase loading (FPU/g glucan)

To elucidate the promoting effect of PP on enzymatic hydrolysis efficiency, enzymatic hydrolysis of PSA-Ba substrate with the addition of PP were further investigated under different conditions. Microcrystalline cellulose (Avicel) was used as a control substrate for enzymatic hydrolysis. As shown in Fig. 3a, the glucose yield of PSA-Ba substrate increased with the increasing addition of PP and reached a plateau when PP addition was increased to 2.5 g/L. In contrast, PP showed no effect on the enzymatic hydrolysis of Avicel regardless of the amount of PP added, indicating no significant interaction between Avicel and PP and/or no apparent inhibition to enzymes. The low glucose yield of Avicel may be mainly ascribed to its higher CrI (Gomide et al. 2019; Ling et al. 2017) than cellulose in PSA-Ba substrate (Additional file 1: Fig. S4). These results illustrate that PP, an protein isolated from inexpensive DPF, was as effective as the widely studied BSA, which can selectively block the lignin on the surface of pretreated substrates and has no interaction with cellulose and enzymes in the hydrolysis medium (Yang and Wyman 2006). This advantage encourages us to further investigate the use of PP as a lignin-blocking additive during enzymatic hydrolysis.

The enzymatic hydrolysis efficiency of PSA-Ba substrate with or without lignin-blocking additive was highly pH-dependent. Without PP addition, the glucose yield of PSA-Ba substrate increased with an increasing pH value from 4.0 to 5.5 of the buffer solution and then decreased slightly at pH = 6 (Fig. 3b). When the pH value of the buffer solution was increased in the range of 4.0 − 5.5, the formation of negative charges (discussed in the following Section) on the surface of lignin and enzymes increased because of the isoelectric point values for lignin sample and cellulase cocktail were less than 5.0 (Wang et al. 2013). The lower isoelectric point than the pH value of the buffer solution favors the generation of intense electrostatic repulsion between lignin and enzymes, thereby alleviating the non-productive adsorption of enzymes on the surface of lignin within the pH range (Lou et al. 2013). Further increase of the pH value of the buffer solution to 6 or higher would denature cellulase and affect the activity of cellulase. After blocking the lignin with PP, the pH of the buffer solution mainly influences the enzyme activity and thereby the glucose yield. This is consistent with the previous report that enzyme from *Trichoderma reesei* has the highest activity in the buffer solution with a pH value of 4.8 − 5.0 (Lan et al. 2013). However, without the addition of PP, the promoting effect of the electrostatic repulsion on enzymatic hydrolysis is not remarkable (glucose yield, 18.9–39.9%, Fig. 3b). Therefore, it is reasonable to believe that non-productive adsorption induced by lignin deposits would be the primary inhibitory mechanism governing the enzymatic hydrolysis efficiency rather than the steric hindrance and pH-caused enzyme activity loss. This speculation was also confirmed by previous studies on non-catalytic proteins as lignin-blocking additives. For example, after adding casein and skim milk into enzymatic hydrolysis systems, the stretching vibrations of feature bonds (C=O and N–H) in proteins absorbed on the surface of DA-pretreated substrate were more evident than those without non-catalytic protein additions (Eckard et al. 2012). Farinas’s (Florencio et al. 2016) and our (Luo et al. 2019) groups also found that the promotion effect of soy protein on the enzymatic hydrolysis of pretreated lignocellulosic substrates was also mainly based on its competitive adsorption with enzymes onto the lignin.

Suppressing non-productive adsorption of lignin to enzymes would substantially reduce the loading of costly enzymes. Without PP addition, the glucose yield of PSA-BA substrate increased from 7.0 to 95.8% with the cellulase loading increasing from 1 to 20 FPU/g glucan (Fig. 3c). With the addition of PP, a glucose yield of 93.6% was achieved at a cellulase loading of only 5 FPU/g glucan. This comparison indicates that the addition of 2.5 g/L PP could reduce the cellulase loading by more than three times (17.5 vs. 5 FPU/g glucan, Fig. 3c) and meanwhile achieve the comparable glucose yields (95.8 vs. 93.6%). Similar to the results shown in Fig. 2, the promoting effect of PP on the PSA-Ba substrate was also comparable to expensive BSA and non-biocompatible Tween 20 and significantly higher than those of LS and PVA (Fig. 3d). Due to the high availability of DPF and simple protein separation method (Fig. 1), the use of the low-cost PP as a lignin-blocking additive to overcome the adverse effect of lignin would be a promising method to reduce the use of costly enzymes and improve the economic feasibility of the biorefining processes involving enzymatic hydrolysis of cellulose.

### Application of PP to other different substrates

**Fig. 4:**
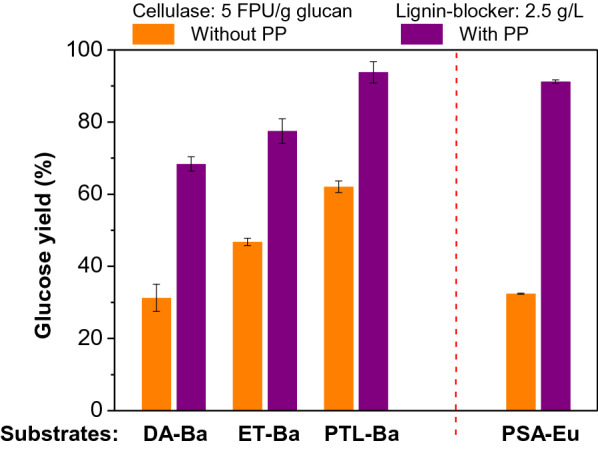
Application of PP to the bamboo and eucalyptus substrates pretreated by different methods. The pH value of buffer solutions and β-glucosidase loading for all enzymatic hydrolysis were 5 and 10 CBU/g glucan, respectively

The performance of PP for improving the enzymatic hydrolysis efficiency of cellulose in other different substrates was further verified. In addition to DA pretreatment, bamboo particles were also pretreated by ET and PTL pretreatments. Under optimized enzymatic hydrolysis conditions (cellulase, 5 FPU/g glucan; lignin-blocking additive, 2.5 g/L; buffer pH value, 5.0), the promotion effect of PP on the PTL-Ba substrate was similar to that of PSA-Ba substrate and better than those of DA-Ba and ET-Ba substrates (Fig. 4). According to the discussion above and previous reports (Ko et al. 2015; Liu et al. 2019; Luo et al. 2019), the differences in the promotion effect was mainly caused by the different enzymatic accessibility of cellulose in the pretreated substrates. With low lignin removal, DA-Ba and ET-Ba substrates had low digestibilities and the promotion effect of PP was not evident.

To further verify the effectiveness of PP towards different lignocelluloses, eucalyptus, a widely studied lignocellulosic feedstock, was also pretreated by PSA pretreatment and used to evaluate the promotion effect of PP. Under the same enzymatic hydrolysis conditions, the addition of PP increased the glucose yield of PSA-Eu substrate from 32.4 to 91.2% (Fig. 4). This result was comparable to those of PSA treated bamboo substrates (Fig. 3), showing good adaptability of PP to different lignocelluloses.

### Mechanism of the promoting effect of PP on enzymatic hydrolysis

**Fig. 5:**
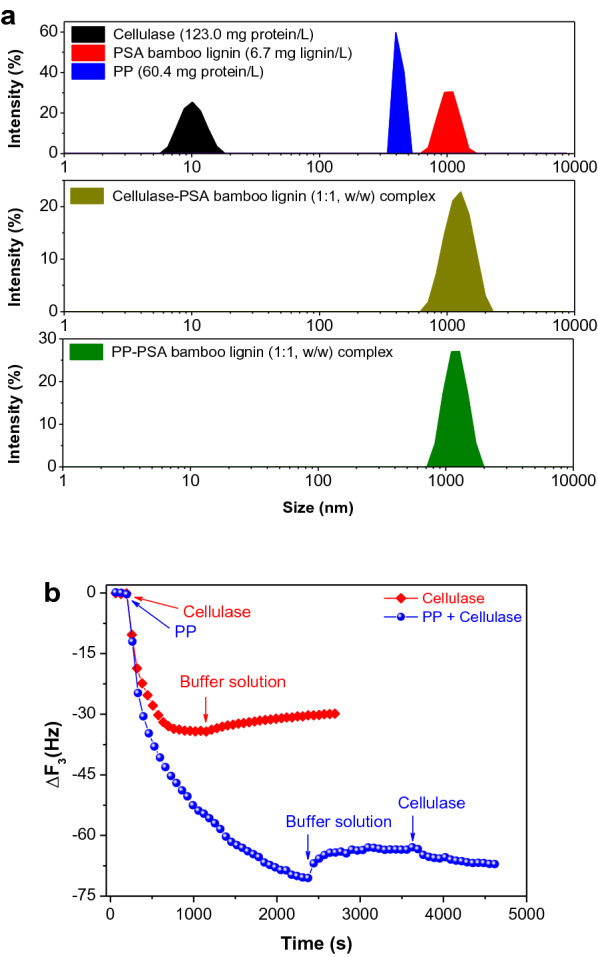
**a** Particle size distributions of cellulase, PP, and PSA supernatants as well as their complexes; **b** adsorption/desorption behaviors of PP and enzymes on the lignin film

The good promoting performance of PP on the enzymatic hydrolysis of pretreated substrates motivates us to explore its mechanism for blocking lignin and reducing non-productive adsorption. To investigate the interaction between protein and lignin, cellulase (123.0 mg/L), PP (60.4 mg/L) and PSA bamboo lignin (6.7 mg/L) supernatants were mixed and the particle sizes before and after mixing were analyzed individually. The average particle sizes were measured to be 10, 396, and 1110 nm for cellulase, PP, and PSA bamboo lignin (Fig. 5a), respectively. After mixing cellulase and PSA bamboo lignin supernatants to obtain a mixture with a lignin/protein mass ratio of 1:1 (w/w) at 25 °C, the peak of cellulase disappeared and a new peak with a larger diameter than that of PSA bamboo lignin itself appeared (Fig. 5a), indicating the formation of a complex via a rapid interaction between lignin and cellulase. Similarly, a PP–PSA bamboo lignin complex (1280 nm) was also readily formed after mixing PP with PSA bamboo lignin. These results reveal that both cellulase and PP can be easily adsorbed on the surface of PSA bamboo lignin, which suggests the feasibility of applying non-catalytic proteins as the alternatives of enzymes to block lignin.

QCM has been regarded as a viable tool to investigate the adsorption/desorption behaviors of enzymes on the lignin films (Lai et al. 2019; Song et al. 2017). The adsorption of cellulase and PP onto PSA bamboo lignin was further quantitatively investigated by QCM. After injecting the cellulase buffer solution (0.5 g/L protein in pH 5 and 50 mmol/L acetate buffer solution) into the QCM flow module with the gold sensor coated with PSA bamboo lignin film and reaching the adsorption equilibrium, the maximum change of QCM frequency (Δ*f*) was 34.3 Hz (Fig. 5b), which corresponds to a maximum adsorption capacity (MAC, ng/cm^2^) of 202.4 ng/cm^2^ based on the Sauerbrey equation (Eq. 3). Injecting PP solution with same protein concentration (0.5 g/L) resulted in a Δ*f* change of 70.5 Hz and a maximum adsorption capacity of 416.0 ng/cm^2^. This comparison suggests that PP had a stronger interaction with PSA bamboo lignin than cellulase. After switching the PP or cellulase buffer solution in QCM flow module to fresh acetate buffer solution (pH 5, 50 mmol/L) to desorb cellulase or PP, the Δ*f* changed from 70.5 to 63.5 Hz for PP and from 34.3 to 29.9 Hz for cellulase, corresponding to only 9.9 and 12.8% desorption of PP and cellulase from the surface of PSA bamboo lignin film (Fig. 5b). In other words, the adsorption of both enzyme and PP on lignin was stable or irreversible in acetate buffer solution, possibly due to strong hydrophobic and/or other interactions. In addition to the blocking effect of PP on lignin, the interaction of the adsorbed PP towards enzyme is also crucial to the enzymatic hydrolysis. When the adsorption of PP onto the lignin film reached equilibrium, the cellulase buffer solution was re-injected into QCM flow module. A Δ*f* change by only 4.1 Hz (63.5–67.6 Hz, Fig. 5b) was observed, indicating that about 88% of cellulase was preserved in the solution after the addition of PP to block lignin. These results illustrate two points: (1) PP demonstrates higher adsorption capacity than cellulase towards lignin, favoring PP to compete with cellulase for blocking lignin; (2) the adsorption of PP onto the lignin film is rather stable, which would be beneficial to the effective blocking of lignin by PP prior to the addition of cellulase. These key points explain the excellent promoting effect of PP on the enzymatic hydrolysis of pretreated substrates. Particularly, the amount of PP added (2.5 g/L) was higher than the amount of enzyme protein (< 0.2 g/L) at an optimized enzyme loading of 5 FPU/g glucan (Fig. 3), which would significantly drive the adsorption between PP and lignin and thereby reduce the adsorption between enzymes and lignin during the enzymatic hydrolysis.

**Fig. 6:**
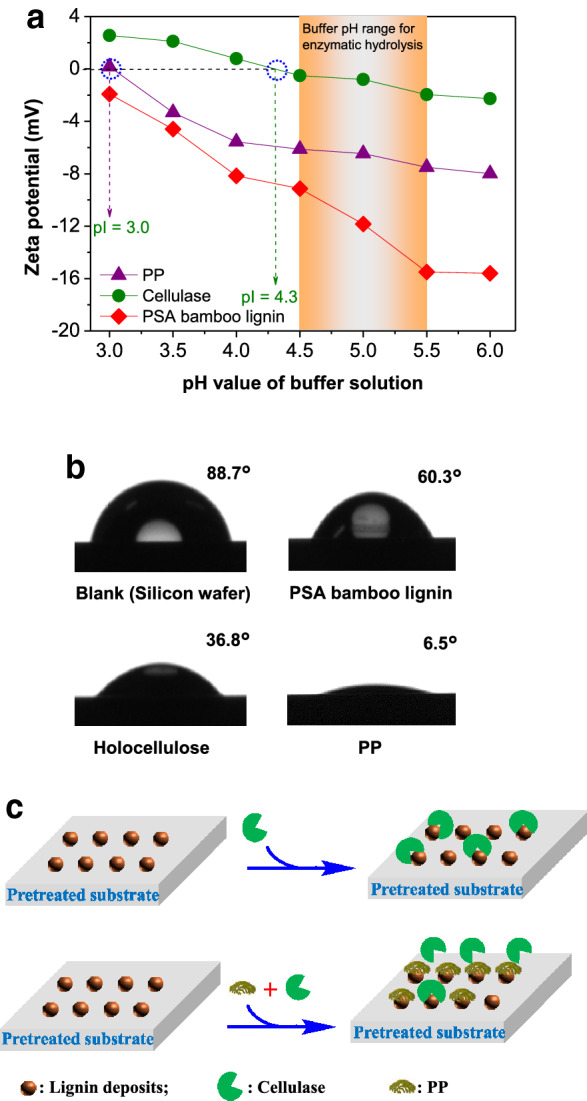
**a** Zeta potentials of PP, cellulase, and PSA bamboo lignin solutions; **b** the contact angles of water on PSA bamboo lignin, holocellulose, and PP films coated on the silicon wafer; **c** a proposed mechanism for the promoting effect of PP on the enzymatic hydrolysis of glucan in pretreated substrates

In view of such robust blocking effects of PP on lignin, the main interactions between protein and lignin were further explored. Proteins including cellulase and non-catalytic proteins are amphipathic macromolecules with different (hydrophilic and hydrophobic) surface areas and charged functional groups (amine and carboxyl groups), and capable of interacting with lignin through hydrophobic, hydrogen-bonding, and/or electrostatic interactions (dos Santos et al. 2019; Li and Zheng 2017). However, the zeta potentials of PSA bamboo lignin (− 9.1 ~ − 15.5 mV), PP (− 6.1 ~ − 7.5 mV), and cellulase (− 0.5 ~ − 2.0 mV) were measured to be negative in the buffer solution within a pH range of 4.5–5.5 that is usually used for enzymatic hydrolysis (Fig. 6a). These negative charges could result in electrostatic repulsion and therefore such an interaction should not significantly contribute to the adsorption of PP to lignin.

Numerous studies have referred hydrophobic interaction as the primary factor governing the adsorption between protein and lignin. Researchers (Ueno et al. 2018) reported that the cellulase molecules could be significantly adsorbed by hydrophobic but uncharged graphite in enzymatic buffer solution, which highly inhibited the hydrolysis of filter paper. This inhibition was effectively overcome with the addition of BSA while PEG had no promotion effect. The researchers stated that the hydrophobic interaction between lignin and BSA would be much stronger than that between lignin and PEG containing plenty of ether linkages in the molecular chains, resulting in almost 90% of the former adsorbed on the hydrophobic graphite while only 10% for the latter. Previous studies also confirmed that the interacting force between hydrophobically modified probe and lignin was measured to be higher than that of hydrophilic one during an atomic force microscope analysis (Qin et al. 2014). Moreover, the hydrophobicity of lignin samples isolated from liquid hot water-pretreated corn stover was also highly related to their abilities to adsorb cellulase (Lu et al. 2016). These conclusions drive us to further investigate the surficial hydrophilicity/hydrophobicity of lignin and PP used in this study. After lignin was spin-coated on the hydrophobic silicon wafer (89° for pure water, Fig. 6b), the contact angle of pure water on the PSA bamboo lignin film was determined to be 60° (Fig. 6b), which was much higher than that of the holocellulose (37°) separated from PSA-Ba substrate, suggesting that the hydrophobicity of PSA bamboo lignin was higher than holocellulose. Since the net surface charges of PP and lignin are negative (Fig. 6a) in the pH range commonly used for enzymatic hydrolysis, in theory, electrostatic interaction will not be the key factor for driving the effective adsorption of PP on the surface of PSA bamboo lignin. Moreover, PP presented much more significant promoting effects on the enzymatic hydrolysis of PSA-Ba substrate covered with hydrophobic lignin deposits than that on the hydrophilic pure cellulose (Fig. 3a). In addition to hydrogen-bonding, the above-mentioned results drive us to speculate that hydrophobic interaction would be one of the most important intermolecular interactions governing the adsorption of PP on the lignin deposits (Figs. 5b and 6b). A relatively low contact angle of water on the PP film (7°) further demonstrates that PP possesses good hydrophilicity. After PP being adsorbed on the hydrophobic lignin surface, a hydration layer will be formed on the surface of PP, inhibiting the non-productive adsorption of cellulase and save the enzyme cost. This promoting effect of PP is similar to the blocking effect of PVP on lignin (Cai et al. 2017a) or the antifouling of marine coatings to shellfish protein under water (Lu et al. 2018).

Consequently, based on above results and discussion, the mechanism shown in Fig. 6c was proposed to illustrate the role of PP in improving enzymatic hydrolysis efficiency. Firstly, with strong adsorption capacity (Fig. 5b), PP can highly compete with cellulase to interact with the lignin deposited on the surface of acid-pretreated substrates through hydrophobic and/or hydrogen-bonding interactions. After PP being adsorbed on the lignin, some functional groups (e.g., amino and hydroxyl groups) on the surface of PP can be further hydrated with water molecules, repelling enzymes from the non-productive adsorption with lignin. PP-induced lignin blocking and enzyme repulsion will lead to more free cellulase molecules to be involved in enzymatic hydrolysis. The promoting effect of low-cost PP would therefore enable decent glucose yields with low enzyme loadings (Fig. 3c), improving the economic efficiency of lignocellulosic saccharification process.

### Effect of PP pre-extraction on the utilization of DPF as a bio-adhesive

**Fig. 7:**
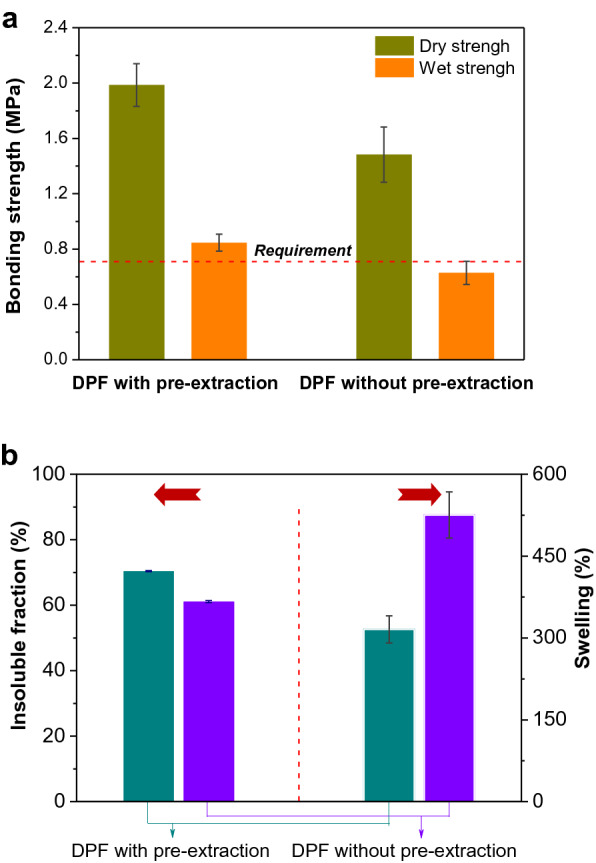
**a** The dry and wet bonding strength of plywoods using DPF as adhesives with and without PP pre-extraction; **b** the insoluble fraction and swelling of DPF-based adhesives with and without PP pre-extraction

To verify the effect of PP extraction on the utilization of DPF, the application of residual DPF as a bio-adhesive for fabricating plywood (Fig. 1) was further investigated. The dry bonding strength of residual DPF-based plywood was higher than DPF-based plywood (Fig. 7a). According to the Chinese National Standard GB/T 9846-2015, residual DPF-based adhesive also resulted in a wet bonding strength of 0.9 MPa that met the requirement of plywood for interior applications while only a wet bonding strength of only 0.6 MPa was obtained for the DPF-based adhesive (Fig. 7a). The insoluble fraction and swelling of residual DPF-based adhesive were lower than those of DPF-based adhesive (Fig. 7b and Additional file 1: Figure S5). We thereby speculate that the better performance of residual DPF-based adhesive should be attributed to the removal of low-molecular-weight and water-soluble proteins (that is PP in the context) (Chen et al. 2015), which would lead to more compact crosslinking structure and higher water resistance of residual DPF-based adhesive.

In order to verify this speculation, the molecular weight of PP extracted by acetate buffer was characterized by SDS-PAGE. The SDS-PAGE measurements of peanut protein isolate (PPI) extracted by a traditional alkali dissolution method and BSA were also used for comparison. SDS-PAGE results showed that the molecular weight (MW) distribution of the proteins in PPI included several regions (Additional file 1: Figure S6): very high (> 50 kDa), high (30–40 kDa), middle (~ 25 kDa), low (10–20 kDa) and very low (< 10 kDa), respectively. However, for different sample loading volumes, the MWs of acetate buffer extracted PP were both lower than that of PPI and BSA, mainly ranging from 10 to 20 kDa (Additional file 1: Figure S6). Therefore, these results supported the speculation of that the pre-extraction mainly separates part of the low-molecular-weight proteins from DPF, which may favor to improve the performance of the residue as an adhesive. Overall, the extraction of PP from DPF can not only provide an effective lignin block additive for promoting enzymatic hydrolysis of cellulose (Figs. 2 and 3), but also is beneficial to the value-added utilization of DPF, making the biorefining processes of woody biomass and DPF more economic.

## Conclusions

This study developed a simple method to processing DPF as an efficient lignin-blocking additive (PP) and a wood adhesive. Biocompatible PPs were used to block lignin that deposited on the surface of pretreated substrates during acidic pretreatments. Due to non-productive adsorption of enzymes onto lignin deposits, high cellulase loadings were required to achieve decent glucose yields. The addition of PP during the enzymatic hydrolysis of pretreated lignocellulosic substrates could significantly reduce the loading of expensive enzymes and meanwhile achieve high glucose yields, greatly improving the economic efficiency of lignocellulose saccharification process. In addition to hydrogen-bonding interaction, hydrophobic force was proposed as the main factor driving the interaction between PP and lignin. Moreover, pre-extraction of the low-molecular-weight and water-soluble PP from DPF could improve the mechanical property and the water resistance of the bio-adhesive synthesized from residual DPF after PP extraction. In view of these advantages, PP would be an ideal alternative of the extensively studied but expensive protein additives such as BSA for blocking lignin deposits, showing great potential for industrial applications.

### Supplementary Information

**Additional file1**: **Figure S1.** SEM images of Un-Ba raw material, DA-Ba and PSA-Ba substrates. The pretreatments conditions used to prepare DA-Ba and PSA-Ba substrates are shown in Table 1. **Figure S2.** C1s in XPS spectra of (a) bamboo raw material and (b) PSA pretreated bamboo substrate. **Figure S3.** The 31P-NMR spectroscopy of PSA bamboo lignin. Figure S4. XRD spectra of PSA pretreated bamboo substrate and corresponding crystallinity index (CrI). **Figure S5.** Bonding strength tests and partial wood failure of plywoods, and swelling of adhesives. **Figure S6.** Sodium dodecyl sulphate–polyacrylamide gel electrophoresis (SDS–PAGE) profiles of PP (lane 1), peanut protein isolate (PPI) (lane 2) and BSA (lane 3). **Table S1.** The concentration of glucose in PP supernatant with and without acid hydrolysis. **Table S2.** The relative amounts of different carbon on the surface of bamboo raw material and PSA pretreated bamboo substrate and corresponding S_lig_. **Table S3.** The contents (mmol/g) of hydroxyl groups on the milled bamboo lignin (BML) and PSA pretreated bamboo lignin.

## Data Availability

All data generated or analyzed during this study are included in this published article and its Additional files.

## Abbreviations

PP: Peanut protein
PPI: Peanut protein isolate
DPF: Defatted peanut flour
PSA: Phenylsulfonic acid
DA: Dilute acid
ET: Ethanol
PEG: Polyethylene glycol
PVP: Polyvinylpyrrolidone
BSA: Bovine serum albumin
PAE: Polyamide-polyamine epichlorohydrin
LS: Lignosulfonate
DLS: Dynamic light scattering
QCM: Quartz crystal microbalance
MAC: Maximum adsorption capacity
BCA: Bicinchoninic acid
Un-Ba: Untreated bamboo particles
DA-Ba: DA-pretreated bamboo
PSA-Ba: PSA-pretreated bamboo

## References

[CR1] Akimkulova A, Zhou Y, Zhao X, Liu D (2016). Improving the enzymatic hydrolysis of dilute acid pretreated wheat straw by metal ion blocking of non-productive cellulase adsorption on lignin. Bioresour Technol.

[CR2] Argyropoulos D, Sun YJ, Paluš E (2002). Isolation of residual kraft lignin in high yield and purity. J Pulp Paper Sci.

[CR3] Bian H, Chen L, Gleisner R, Dai H, Zhu JY (2017). Producing wood-based nanomaterials by rapid fractionation of wood at 80 °C using a recyclable acid hydrotrope. Green Chem.

[CR4] Börjesson J, Engqvist M, Sipos B, Tjerneld F (2007). Effect of poly(ethylene glycol) on enzymatic hydrolysis and adsorption of cellulase enzymes to pretreated lignocellulose. Enzyme Microb Tech.

[CR5] Cai C, Qiu X, Zeng M, Lin M, Lin X, Lou H, Zhan X, Pang Y, Huang J, Xie L (2017). Using polyvinylpyrrolidone to enhance the enzymatic hydrolysis of lignocelluloses by reducing the cellulase non-productive adsorption on lignin. Bioresour Technol.

[CR6] Cai C, Zhan X, Zeng M, Lou H, Pang Y, Yang J, Yang D, Qiu X (2017). Using recyclable pH-responsive lignin amphoteric surfactant to enhance the enzymatic hydrolysis of lignocelluloses. Green Chem.

[CR7] Chen L, Dou J, Ma Q, Li N, Wu R, Bian H, Yelle DJ, Vuorinen T, Fu S, Pan X, Zhu J (2017). Rapid and near-complete dissolution of wood lignin at ≤80°C by a recyclable acid hydrotrope. Sci Adv.

[CR8] Chen N, Zeng Q, Lin Q, Rao J (2015). Development of defatted soy flour based bio-adhesives using Viscozyme L. Ind Crop Prod.

[CR9] Donohoe BS, Decker SR, Tucker MP, Himmel ME, Vinzant TB (2008). Visualizing lignin coalescence and migration through maize cell walls following thermochemical pretreatment. Biotechnol Bioeng.

[CR10] dos Santos AC, Ximenes E, Kim Y, Ladisch MR (2019). Lignin–enzyme interactions in the hydrolysis of lignocellulosic biomass. Trends Biotechnol.

[CR11] Eckard AD, Muthukumarappan K, Gibbons W (2012). Analysis of casein biopolymers adsorption to lignocellulosic biomass as a potential cellulase stabilizer. J Biomed Biotechnol.

[CR12] Eriksson T, Börjesson J, Tjerneld F (2002). Mechanism of surfactant effect in enzymatic hydrolysis of lignocellulose. Enzyme Microb Tech.

[CR13] Florencio C, Badino AC, Farinas CS (2016). Soybean protein as a cost-effective lignin-blocking additive for the saccharification of sugarcane bagasse. Bioresour Technol.

[CR14] Gomide FTF, da Silva ASA, da Silva Bon EP, Alves TLM (2019). Modification of microcrystalline cellulose structural properties by ball-milling and ionic liquid treatments and their correlation to enzymatic hydrolysis rate and yield. Cellulose.

[CR15] Himmel ME, Ding S-Y, Johnson DK, Adney WS, Nimlos MR, Brady JW, Foust TD (2007). Biomass recalcitrance: engineering plants and enzymes for biofuels production. Science.

[CR16] Klein-Marcuschamer D, Oleskowicz-Popiel P, Simmons BA, Blanch HW (2012). The challenge of enzyme cost in the production of lignocellulosic biofuels. Biotechnol Bioeng.

[CR17] Ko JK, Kim Y, Ximenes E, Ladisch MR (2015). Effect of liquid hot water pretreatment severity on properties of hardwood lignin and enzymatic hydrolysis of cellulose. Biotechnol Bioeng.

[CR18] Kristensen JB, Börjesson J, Bruun MH, Tjerneld F, Jørgensen H (2007). Use of surface active additives in enzymatic hydrolysis of wheat straw lignocellulose. Enzyme Microb Tech.

[CR19] Lai C, Yang B, Lin Z, Jia Y, Huang C, Li X, Song X, Yong Q (2019). New strategy to elucidate the positive effects of extractable lignin on enzymatic hydrolysis by quartz crystal microbalance with dissipation. Biotechnol Biofuels.

[CR20] Lan TQ, Lou H, Zhu JY (2013). Enzymatic saccharification of lignocelluloses should be conducted at elevated pH 5.2–6.2. BioEnerg Res.

[CR21] Lan TQ, Wang SR, Li H, Qin YY, Yue GJ (2020). Effect of lignin isolated from p-toluenesulfonic acid pretreatment liquid of sugarcane bagasse on enzymatic hydrolysis of cellulose and cellulase adsorption. Ind Crop Prod.

[CR22] Li X, Zheng Y (2017). Lignin-enzyme interaction: mechanism, mitigation approach, modeling, and research prospects. Biotechnol Adv.

[CR23] Ling Z, Chen S, Zhang X, Takabe K, Xu F (2017). Unraveling variations of crystalline cellulose induced by ionic liquid and their effects on enzymatic hydrolysis. Sci Rep.

[CR24] Liu H, Sun J, Leu S-Y, Chen S (2016). Toward a fundamental understanding of cellulase-lignin interactions in the whole slurry enzymatic saccharification process. Biofuels Bioprod Bior.

[CR25] Liu H, Zhu JY, Fu SY (2010). Effects of lignin−metal complexation on enzymatic hydrolysis of cellulose. J Agric Food Chem.

[CR26] Liu J, Hu H, Gong Z, Yang G, Li R, Chen L, Huang L, Luo X (2019). Near-complete removal of non-cellulosic components from bamboo by 1-pentanol induced organosolv pretreatment under mild conditions for robust cellulose enzymatic hydrolysis. Cellulose.

[CR27] Lou H, Zhu JY, Lan TQ, Lai H, Qiu X (2013). pH-induced lignin surface modification to reduce nonspecific cellulase binding and enhance enzymatic saccharification of lignocelluloses. Chemsuschem.

[CR28] Lu Q, Huang J, Maan O, Liu Y, Zeng H (2018). Probing molecular interaction mechanisms of organic fouling on polyamide membrane using a surface forces apparatus: implication for wastewater treatment. Sci Total Environ.

[CR29] Lu X, Zheng X, Li X, Zhao J (2016). Adsorption and mechanism of cellulase enzymes onto lignin isolated from corn stover pretreated with liquid hot water. Biotechnol Biofuels.

[CR30] Luo X, Gong Z, Shi J, Chen L, Zhu W, Zhou Y, Huang L, Liu J (2020). Integrating benzenesulfonic acid pretreatment and bio-based lignin-shielding agent for robust enzymatic conversion of cellulose in bamboo. Polymers.

[CR31] Luo X, Liu J, Zheng P, Li M, Zhou Y, Huang L, Chen L, Shuai L (2019). Promoting enzymatic hydrolysis of lignocellulosic biomass by inexpensive soy protein. Biotechnol Biofuels.

[CR32] Öhgren K, Bura R, Saddler J, Zacchi G (2007). Effect of hemicellulose and lignin removal on enzymatic hydrolysis of steam pretreated corn stover. Bioresour Technol.

[CR33] Österberg M (2000). The effect of a cationic polyelectrolyte on the forces between two cellulose surfaces and between one cellulose and one mineral surface. J Colloid Interf Sci.

[CR34] Pan X, Arato C, Gilkes N, Gregg D, Mabee W, Pye K, Xiao Z, Zhang X, Saddler J (2005). Biorefining of softwoods using ethanol organosolv pulping: preliminary evaluation of process streams for manufacture of fuel-grade ethanol and co-products. Biotechnol Bioeng.

[CR35] Qin C, Clarke K, Li K (2014). Interactive forces between lignin and cellulase as determined by atomic force microscopy. Biotechnol Biofuels.

[CR36] Ragauskas AJ, Williams CK, Davison BH, Britovsek G, Cairney J, Eckert CA, Frederick WJ, Hallett JP, Leak DJ, Liotta CL, Mielenz JR, Murphy R, Templer R, Tschaplinski T (2006). The path forward for biofuels and biomaterials. Science.

[CR37] Saini JK, Patel AK, Adsul M, Singhania RR (2016). Cellulase adsorption on lignin: a roadblock for economic hydrolysis of biomass. Renew Energ.

[CR38] Sannigrahi P, Kim DH, Jung S, Ragauskas A (2011). Pseudo-lignin and pretreatment chemistry. Energ Environ Sci.

[CR39] Sauerbrey G (1959). Verwendung von schwingquarzen zur wägung dünner schichten und zur mikrowägung. Z Phys.

[CR40] Selig MJ, Viamajala S, Decker SR, Tucker MP, Himmel ME, Vinzant TB (2007). Deposition of lignin droplets produced during dilute acid pretreatment of maize stems retards enzymatic hydrolysis of cellulose. Biotechnol Progr.

[CR41] Shi H, Zhou M, Li C, Sheng X, Yang Q, Li N, Niu M (2019). Surface sediments formation during auto-hydrolysis and its effects on the benzene-alcohol extractive, absorbability and chemical pulping properties of hydrolyzed acacia wood chips. Bioresour Technol.

[CR42] Shuai L, Amiri MT, Questell-Santiago YM, Héroguel F, Li Y, Kim H, Meilan R, Chapple C, Ralph J, Luterbacher JS (2016). Formaldehyde stabilization facilitates lignin monomer production during biomass depolymerization. Science.

[CR43] Shuai L, Questell-Santiago YM, Luterbacher JS (2016). A mild biomass pretreatment using γ-valerolactone for concentrated sugar production. Green Chem.

[CR44] Sluiter A, Hames B, Ruiz R, Scarlata C, Sluiter J, Templeton D, Crocker D (2012) Determination of structural carbohydrates and lignin in biomass. Laboratory Analytical Procedure (LAP), Technical Report: NREL/TP-510–42618. National Renewable Energy Laboratory, Golden, Colorado

[CR45] Smith PK, Krohn RI, Hermanson GT, Mallia AK, Gartner FH, Provenzano MD, Fujimoto EK, Goeke NM, Olson BJ, Klenk DC (1985). Measurement of protein using bicinchoninic acid. Anal Biochem.

[CR46] Song J, Yang F, Zhang Y, Hu F, Wu S, Jin Y, Guo J, Rojas OJ (2017). Interactions between fungal cellulases and films of nanofibrillar cellulose determined by a quartz crystal microbalance with dissipation monitoring (QCM-D). Cellulose.

[CR47] Sun Y, Cheng JJ (2005). Dilute acid pretreatment of rye straw and Bermudagrass for ethanol production. Bioresour Technol.

[CR48] Ueno Y, Taneda D, Okino S, Shirai Y (2018). Inhibitory effect of additives on cellulase adsorption mediated by hydrophobic interaction. J Jpn Petrol Inst.

[CR49] Wang Z, Zhu JY, Fu Y, Qin M, Shao Z, Jiang J, Yang F (2013). Lignosulfonate-mediated cellulase adsorption: enhanced enzymatic saccharification of lignocellulose through weakening nonproductive binding to lignin. Biotechnol Biofuels.

[CR50] Yang B, Wyman CE (2004). Effect of xylan and lignin removal by batch and flowthrough pretreatment on the enzymatic digestibility of corn stover cellulose. Biotechnol Bioeng.

[CR51] Yang B, Wyman CE (2006). BSA treatment to enhance enzymatic hydrolysis of cellulose in lignin containing substrates. Biotechnol Bioeng.

[CR52] Yu H, Xu Y, Hou J, Nie S, Liu S, Wu Q, Liu Y, Liu Y, Yu S (2020). Fractionation of corn stover for efficient enzymatic hydrolysis and producing platform chemical using p-toluenesulfonic acid/water pretreatment. Ind Crop Prod.

[CR53] Zheng L, Zhao Y, Xiao C, Sun-Waterhouse D, Zhao M, Su G (2015). Mechanism of the discrepancy in the enzymatic hydrolysis efficiency between defatted peanut flour and peanut protein isolate by Flavorzyme. Food Chem.

[CR54] Zheng P, Chen N, Mahfuzul Islam SM, Ju L-K, Liu J, Zhou J, Chen L, Zeng H, Lin Q (2019). Development of self-cross-linked soy adhesive by enzyme complex from Aspergillus niger for production of all-biomass composite materials. ACS Sustain Chem Eng.

[CR55] Zheng P, Zeng Q, Lin Q, Fan M, Zhou J, Rao J, Chen N (2019). Investigation of an ambient temperature-curable soy-based adhesive for wood composites. Int J Adhes Adhes.

