# Supplementary material for: Understanding the promoting effect of non-catalytic protein on enzymatic hydrolysis efficiency of lignocelluloses
Source: Bioresour Bioprocess. 2021 Jan 29;8(1):9. doi: 10.1186/s40643-021-00363-9 (PMC10991106; doi:10.1186/s40643-021-00363-9)
Supplement: Supplementary file 1 — Additional file1: Figure S1. SEM images of Un-Ba raw material, DA-Ba and PSA-Ba substrates. The pretreatments conditions used to prepare DA-Ba and PSA-Ba substrates are shown in Table 1. Figure S2. C1s in XPS spectra of (a) bamboo raw material and (b) PSA pretreated bamboo substrate. Figure S3. The 31P-NMR spectroscopy of PSA bamboo lignin. Figure S4. XRD spectra of PSA pretreated bamboo substrate and corresponding crystallinity index (CrI). Figure S5. Bonding strength tests and partial wood failure of plywoods, and swelling of adhesives. Figure S6. Sodium dodecyl sulphate–polyacrylamide gel electrophoresis (SDS–PAGE) profiles of PP (lane 1), peanut protein isolate (PPI) (lane 2) and BSA (lane 3). Table S1. The concentration of glucose in PP supernatant with and without acid hydrolysis. Table S2. The relative amounts of different carbon on the surface of bamboo raw material and PSA pretreated bamboo substrate and corresponding Slig. Table S3. The contents (mmol/g) of hydroxyl groups on the milled bamboo lignin (BML) and PSA pretreated bamboo lignin. [file 40643_2021_363_MOESM1_ESM.docx]

**Understanding the promoting effect of non-catalytic protein on enzymatic hydrolysis efficiency of lignocelluloses**

Zhenggang Gong^1, †^, Guangxu Yang^1, †^, Junlong Song^2^, Peitao Zheng^3^, Jing Liu^1^, Wenyuan Zhu^2^, Liulian Huang^1^, Lihui Chen^1^, Xiaolin Luo^1, 2, *^ and Li Shuai^1,^ *

^1^ College of Materials Engineering, Fujian Agriculture and Forestry University, Fuzhou 350002, China

^2^ Jiangsu Provincial Key Laboratory of Pulp and Paper Science and Technology, Nanjing Forestry University, Nanjing 210037, China

^3^ Department of Materials Science & Engineering, Southern University of Science and Technology, Shenzhen 518055, China

^†^ These two authors contributed equally to this work.

***** Correspondence: xluo@fafu.edu.cn (X. L.); lishuai@fafu.edu.cn (L. S.).

**Supporting information**

**Un-Ba**









**DA-Ba**









**PSA-Ba**









**Figure S1.** SEM images of Un-Ba raw material, DA-Ba and PSA-Ba substrates. The pretreatments conditions used to prepare DA-Ba and PSA-Ba substrates are shown in Table 1.

**Figure S2.** C1s in XPS spectra of (a) bamboo raw material and (b) PSA pretreated bamboo substrate.


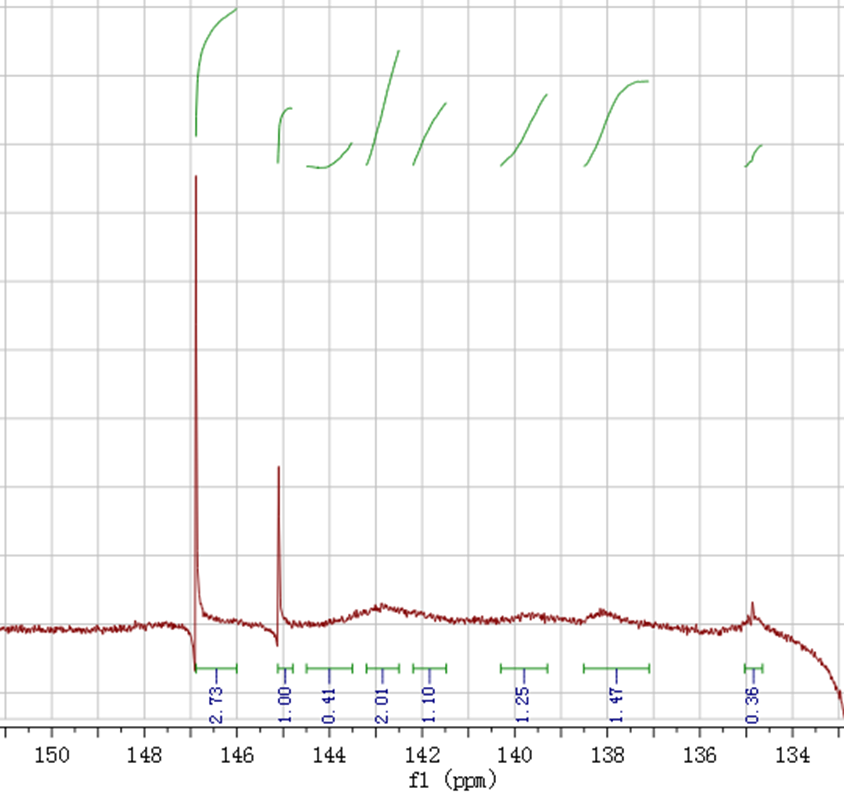


**Figure S3.** The ^31^P-NMR spectroscopy of PSA bamboo lignin.

**Figure S4.** XRD spectra of PSA pretreated bamboo substrate and corresponding crystallinity index (CrI). The CrI data of bamboo raw material was adopted from our previous report (*Cellulose, 2019, 6, 3801–3814*).


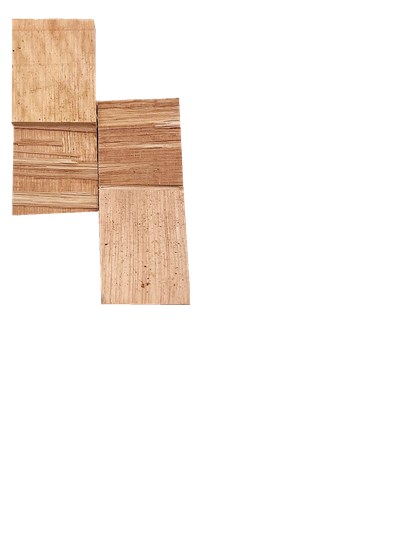

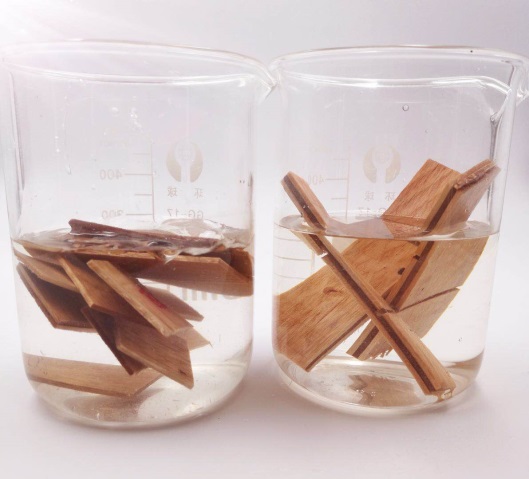

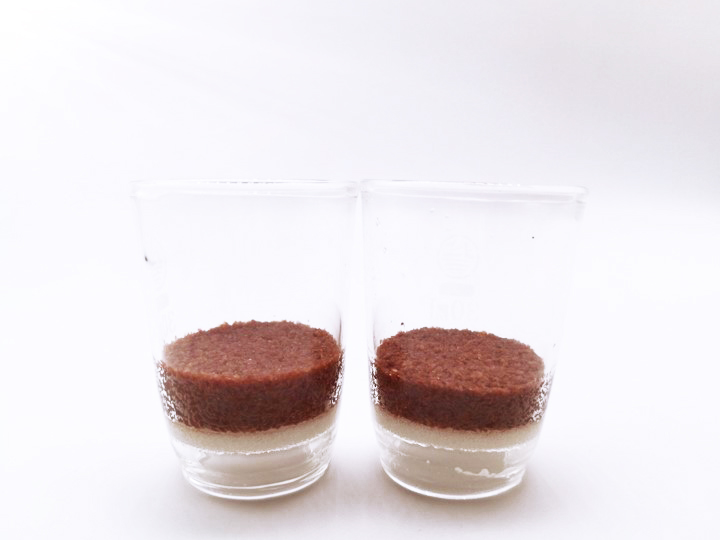

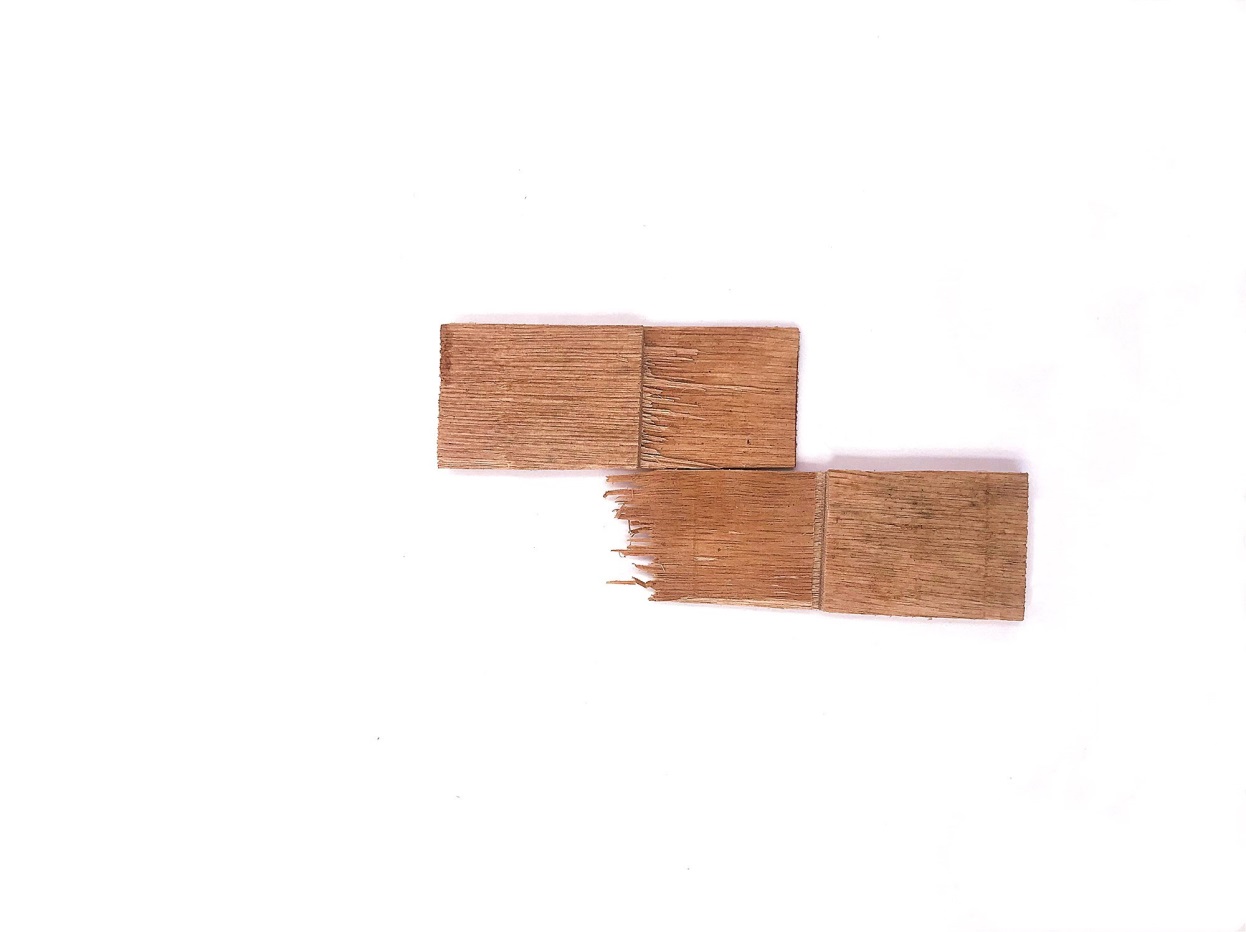


**Pre-soaking of plywood for wet bonding test**

**Swelling measurements of DPF-based adhesives**

**Wood failure**

**Specimens**

**Figure S5.** Bonding strength tests and partial wood failure of plywoods, and swelling of adhesives.


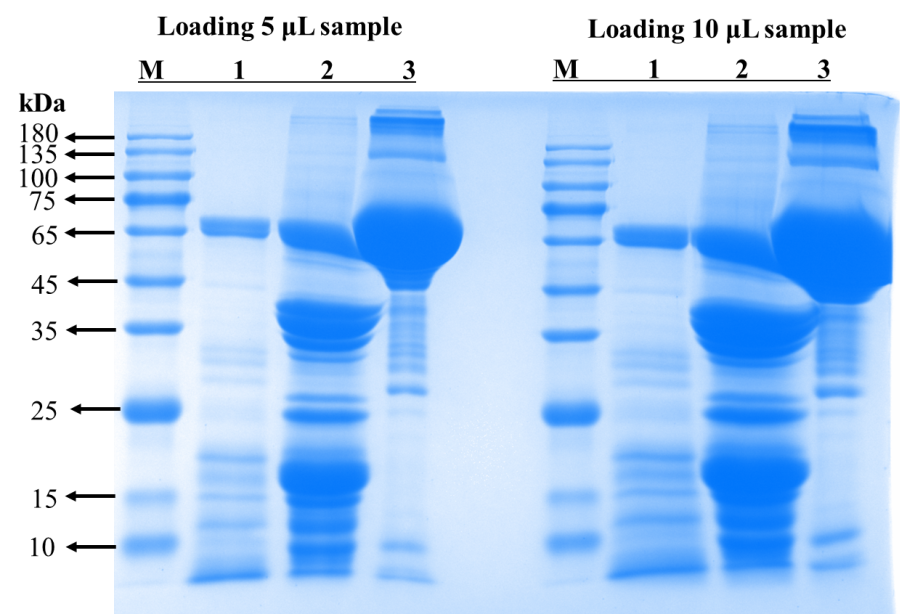


**Figure S6.** Sodium dodecyl sulphate–polyacrylamide gel electrophoresis (SDS–PAGE) profiles of PP (lane 1), peanut protein isolate (PPI) (lane 2) and BSA (lane 3). Lane M is the standard protein markers (M5 Prestained Protein Ladder, 10-180 kDa).

**Table S1** The concentration of glucose in PP supernatant with and without acid hydrolysis.

|  | Concentration of glucose in PP supernatant (mg/L) | Dilution times^b^ | Interference to glucose measurement (%)^c^ |
| --- | --- | --- | --- |
| Without acid hydrolysis | 8.5 | 3.4 | 0.03 |
| With acid hydrolysis^a^ | 36.6 | 3.4 | 0.12 |

^a^ Adding concentrated acid into peanut protein supernatant to obtain an sulfuric acid content of 4 wt% and then hydrolyzing resultant solution at 121°C for 1 h.

^b^ This refers to the dilution times of peanut protein supernatant after adding buffer solution to enzymatic hydrolysis system.

^c^ The interference of the glucose in the peanut protein supernatant to enzymatic hydrolysis efficiency was calculated based on the measured concentration of glucose in enzymatic hydrolysate using DA-Ba substrate and 2.5 g/L PP. The data are the percentage of the concentration of glucose from PP supernatant to the measured total glucose concentration after 72-h enzymatic hydrolysis.

**Table S2** The relative amounts of different carbon on the surface of bamboo raw material and PSA pretreated bamboo substrate and corresponding S_lig_.

| Samples | Elements contents (wt %) | | | |  | | |
| --- | --- | --- | --- | --- | --- | --- | --- |
|  | C1s Scan A | C1s Scan B | C1s Scan C | O1s | | O/C | S_lig_^b^ (%) |
|  | C-C, C-H | C-O | C=O, O-C-O |  |  |  |  |
| Bamboo raw material^a^ | 19.1 | 37.9 | 7.1 | 35.9 | | 0.56 | 53.8 |
| PSA pretreated bamboo substrate | 24.5 | 36.4 | 7.5 | 31.7 | | 0.46 | 73.4 |

^a^ The data of bamboo raw material was adopted from our previous report (*Cellulose, 2019, 6, 3801–3814*).

^b^ S_lig_ refers to the surface lignin coverage (%).

**Table S3** The contents (mmol/g) of hydroxyl groups on the milled bamboo lignin (BML) and PSA pretreated bamboo lignin.

| Functional groups | Milled bamboo lignin (BML)^a^ | PSA pretreated bamboo lignin |
| --- | --- | --- |
| Aliphatic OH | 0.95 | 0.35 |
| Condensed syringyl phenolic hydroxyls (CS) | 0.03 | 0.05 |
| Non-condensed syringyl phenolic hydroxyls (NS) | 0.06 | 0.26 |
| Condensed guaiacyl phenolic hydroxyls (CG) | 0.05 | 0.14 |
| Non-condensed guaiacyl phenolic hydroxyls (NG) | 0.09 | 0.16 |
| p-hydroxybenzoate phenolic hydroxyls (PB-OH) | 0.02 | 0.19 |
| Carboxylic groups (COOH) | 0.20 | 0.05 |
| Total phenolic OH | 0.26 | 0.81 |

^a^ The data of bamboo raw material was adopted from our previous report (*Cellulose, 2019, 6, 3801–3814*).
